# An Improved Strategy for Generating Forces in Steered Molecular Dynamics: The Mechanical Unfolding of Titin, e2lip3 and Ubiquitin

**DOI:** 10.1371/journal.pone.0013068

**Published:** 2010-09-29

**Authors:** Bosco K. Ho, David A. Agard

**Affiliations:** Howard Hughes Medical Institute and the Department of Biophysics and Biochemistry, University of California San Francisco, San Francisco, California, United States of America; University of Wales Bangor, United Kingdom

## Abstract

One of the applications of Molecular Dynamics (MD) simulations is to explore the energetic barriers to mechanical unfolding of proteins such as occurs in response to the mechanical pulling of single molecules in Atomic Force Microscopy (AFM) experiments. Although Steered Molecular Dynamics simulations have provided microscopic details of the unfolding process during the pulling, the simulated forces required for unfolding are typically far in excess of the measured values. To rectify this, we have developed the Pulsed Unconstrained Fluctuating Forces (PUFF) method, which induces constant-momentum motions by applying forces directly to the instantaneous velocity of selected atoms in a protein system. The driving forces are applied in pulses, which allows the system to relax between pulses, resulting in more accurate unfolding force estimations than in previous methods. In the cases of titin, ubiquitin and e2lip3, the PUFF trajectories produce force fluctuations that agree quantitatively with AFM experiments. Another useful property of PUFF is that simulations get trapped if the target momentum is too low, simplifying the discovery and analysis of unfolding intermediates.

## Introduction

Many crucial biological processes occur through large conformational changes in proteins, such as the unfolding of titin in the muscle sarcomere. The ability to model mechanical forces in such processes provides an understanding of how large conformational changes occur in microscopic detail. Although Molecular Dynamics (MD) simulations are generally accepted to accurately model protein dynamics [1], commonly available hardware can only simulate systems for hundreds of nanoseconds (although a few microsecond simulations have been reported [2], [3]. Unfortunately, many important biological processes, especially those accompanied by large conformational changes, take place on timescales of milliseconds to seconds. In order to see such large conformational changes, various techniques have been used to augment MD with improved sampling methods, such as replica-exchange [4], and methods that directly induce conformational change.

There are two broad cases used in directly inducing conformation change in MD. When a reaction pathway (typically starting and ending states) has already been determined, Targeted MD, or umbrella sampling, uses harmonic restraints to sample pre-defined intermediate conformations along the pathway [5]. In cases where only a starting conformation is known, several different types of force-inducing protocols have been used to generate pathways from a starting conformation. RMSD potentials can be used to generate low energy pathways away from the starting state by using increasing RMSD as a driving force [6]–[8]. In processes such as the unfolding of titin by mechanical stress, where there is an obvious force to be applied to the starting conformation, Steered MD can generate new trajectories by setting pre-defined moving harmonic distance restraints to force the system away from the starting configuration along a defined vector [9]. Steered MD has been used to explore systems such as the rotation of the gamma-subunit of ATPase [10]–[11], and the unfolding of fibronectin [12].

Recent developments in Atomic Force Microscopy (AFM) have provided quantitative experiments that measure the response of single molecules to pulling forces applied to defined sites within the molecule. One very well studied system is the I27 domain of titin, an immunoglobin domain. In the constant-velocity AFM pulling experiments of I27, unfolding forces of 150–300 pN were measured at a pulling velocity of 10^−8^ Å/ps [13]. The average unfolding force was found to be dependent on the pulling velocity over a range of 10^−10^–10^−7^ Å/ps [14]. In another study, force-clamp AFM experiments found a peak unfolding force of 180 pN [15], which provides a good single value for the unfolding force. Other experiments have identified an unfolding intermediate that occurs at forces of 60–150 pN, with an averaged F_intermediate_ = 100 pN, and an extension of ∼10 Å [16]–[17] [15].

The AFM experiments of I27 provide a comprehensive set of data to compare with simulation. In order to explore the mechanical pulling of I27 on the microscopic level, Steered MD simulations induce a constant-velocity motion by applying moving harmonic restraints to defined atoms or groups within the protein [18], [19]. However, given the practical limitations of simulation timescales, Steered MD studies are typically performed at pulling velocities of ∼1 Å/ps which is typically 10^8^ orders of magnitude greater than used in the experimental AFM studies. Although such studies were useful for revealing atomic-level details involved in the unfolding of titin, the forces generated by the harmonic springs in Steered MD (∼1000 pN) were far in excess of the 180 pN measured in the AFM experiments.

In order to overcome the problem of generating forces with harmonic restraints, we have developed a force-inducing protocol that is conceptually different than Steered MD. This protocol, which we call Pulsed Unconstrained Fluctuating Forces (PUFF), generates force pulses that directly control the instantaneous velocity of defined locations within the protein and then allows them to relax. In PUFF, forces are applied directly to the instantaneous velocities of atoms, without the need for intermediate harmonic restraints. This results in direct control of the magnitude of the applied forces. If the restrained groups are moving too fast, PUFF will slow them down and vice versa, thus damping velocity fluctuations. As the PUFF forces are applied intermittently, the system is allowed to respond to or resist the applied forces. One interesting consequence is that the system can get trapped, which provides an easy way to identify unfolding intermediates and the critical forces that are needed to induce conformational change. The use of pulses was first developed in a protocol that generates local perturbations in proteins using sidechain rotamers [20].

Using the PUFF protocol on the I27 domain of titin, we show that it is possible to generate unfolding trajectories with unfolding forces that compare well with the AFM measurements and that are much lower than those deduced from standard simulations. We further show that PUFF quantitatively accounts for the measured differences in critical forces when using different pulling geometries in both e2lip3 [21] and ubiquitin [22].

## Results

### Applying PUFF to the unfolding of titin

**Figure 1:**
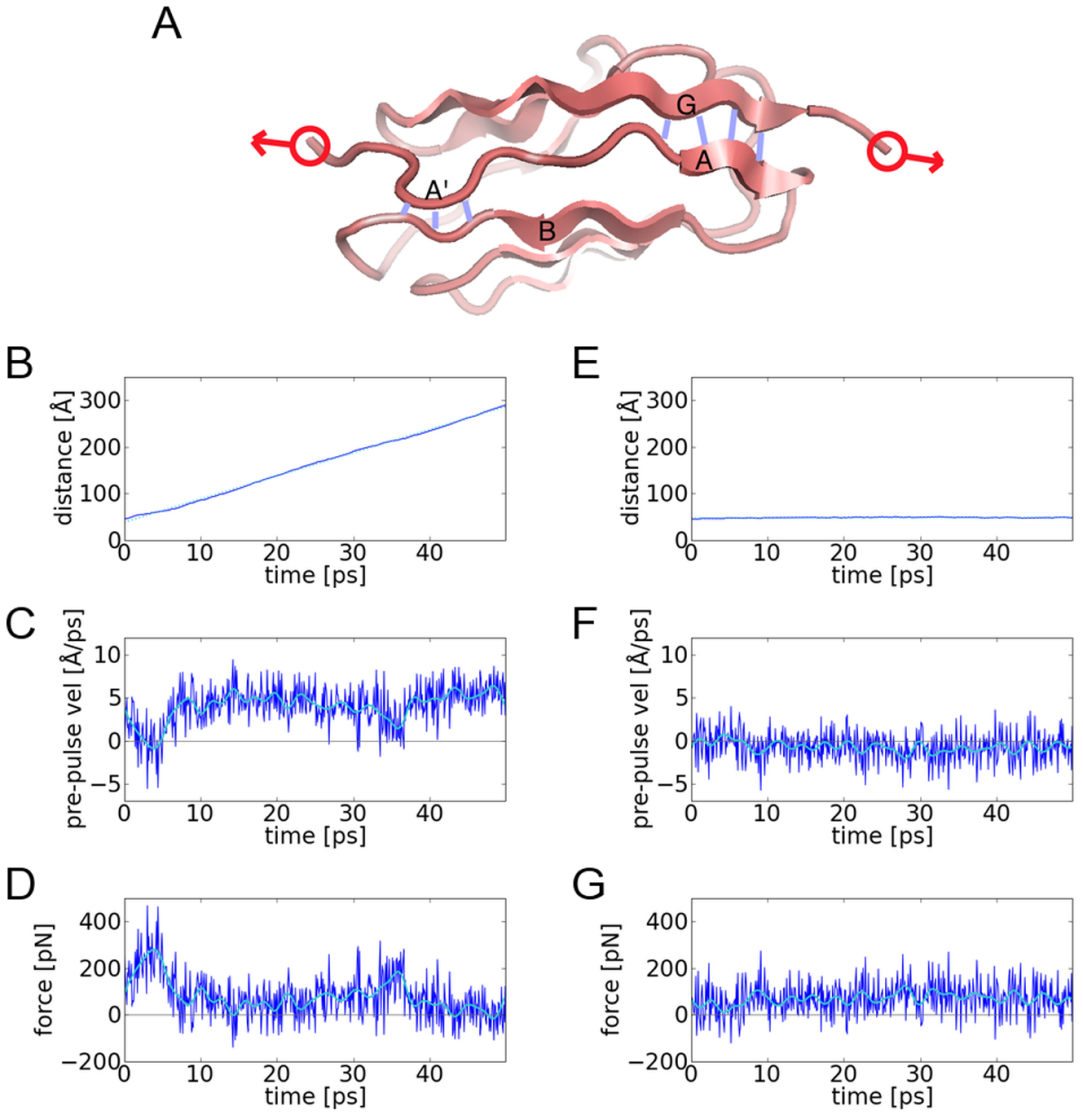
Trajectories of pulling the I27 domain of titin with constant-momentum. (A) Schematic of I27 for target pulling [1TIT]. The key interactions for the unfolding intermediate are the hydrogen bonds (blue) between β-strand-A' and β-strand-B, and between β-strand-A and β-strand-G. In the pulling experiments and simulations, the anchor points for the pulling are the N and C terminii (red). (B) The trajectory for a target velocity of 6.0 Å/ps shows constant velocity motion with a fitted slope of 5.9 Å/ps. (C) The pre-pulse velocities fluctuate around 4.1 Å/ps except for the early part of the trajectory where the velocities is close to zero. (D) The applied forces derived from the change in velocities from the pre-pulse velocities to the target velocity (dark blue). As the forces fluctuate ∼150 pN, to find the general shape of the curve (light blue), a low-pass FFT filter was used to filter out the fluctuations. The fitted curve has a maximum of 280 pN near the beginning of the trajectory before dropping down to ∼20 pN. In the second column are the results for the trajectory with a target velocity of 1.00 Å/ps. (E) The system is effectively trapped as the distance between the anchor points do not change. (F) The pre-pulse velocities are negative −0.7 Å/ps, due to the reflection against the free-energy barrier. (G) The applied forces. The fitted curve has a maximum of 93 pN that is maintained throughout the simulation.

We first use PUFF to explore the mechanical unfolding of the titin I27 domain. As in the AFM experiment (Figure 1A), the N- and C-terminal residues are pulled apart, except here, a constant momentum restraint is used to achieve a desired separation velocity (V_target_). The run is broken into 100 fs pulses and at the beginning of each pulse, PUFF applies a force by setting the instantaneous velocity of the atoms in the N terminal residue to −0.5 V_target_ and the atoms in the C terminal residue to +0.5 V_target_. The exact applied force is defined by the change in velocity needed to bring the pre-pulse velocity to the target velocity V_target_ (see Methods). As the velocity is reset to V_target_ at the beginning of each pulse, the momentum is effectively fixed at the beginning of each pulse, which caps the amount of force applied. Thus the V_target_ defines a constant momentum. In the next section, the relationship between the target velocity V_target_ and the maximal applied force will be derived.

Pulling with V_target_ = 6.0 Å/ps unfolds the I27 domain without any significant barriers even in a quite short 50 ps simulation. As measured by the distance between the center of masses of the two anchor groups, the two terminii are observed to separate at an approximately constant velocity (slope = 5.9 Å/ps, Figure 1B). As this is close to the target velocity of 6.0 Å/ps, the motion does not suffer any great impedance. At the end of the simulation at 50 ps, the protein I27 is fully extended with an end-to-end distance of ∼300 Å.

To examine how the instantaneous velocities evolve over time, the velocities at the end of one relaxation period and just before the application of the force at the beginning of the next pulse are shown in Figure 1C. Pre-pulse velocities significantly lower than the target velocity of +6.0 Å/ps indicate that the system has resisted the application of force in the last pulse. In this case, the system resists the force in the first 5 ps, as the pre-pulse velocities dip to zero, but after 10 ps, there is little resistance as the pre-pulse velocities rise to an average of ∼5 Å/ps. As the force is applied at the instant between pulses, the force is equal to the change in momentum, which is mass times the difference between the pre-pulse velocitiy and the target velocity (Figure 1D). There is a burst of force with a maximum of 280 pN in the first 5 ps, after which, much smaller forces are needed to maintain the motion defined by the target velocity of +6.0 Å/ps.

In a second example, the I27 domain is pulled with a constant-momentum at a target velocity of V_target_ = 1.0 Å/ps. At this target velocity the protein is trapped in the folded state and the end-to-end distance remains at a constant 50 Å throughout the simulation (Figure 1E). The pre-pulse velocity has an average negative value of -0.7 Å/ps (Figure 1F), representing a strong restoring force that evolves counter to the applied pulse during the relaxation period. As the velocity is set to the target velocity at the next instant, which is the beginning of the next pulse, the pre-pulse and target velocities represent the instantaneous velocities at the boundaries of a given pulse. During an average pulse, the velocity fluctuates between 1.0 Å/ps and −0.70 Å/ps, and the protein moves back-and-forth over a short distance. A simple estimate for this distance is V_max_×T_pulse_ = 1.0 Å/ps×100 fs = 0.1 Å. The force-time curve (Figure 1G), shows that the average force is ∼90 pN indicating that at a target velocity of 1.0 Å/ps, the ∼90 pN generated at the beginning of a pulse, is insufficient to break the I27 domain out of the folded minimum. Since the protein is trapped, the maximum amount of force is generated at every pulse, which is continually being resisted.

In the analysis of PUFF forces, it can thus be seen that an average negative pre-pulse velocity indicates that the system is resisting the applied force to remain in the folded state. Comparing the two trajectories, it is apparent that the minimum force required to break out of the folded state lies somewhere between 90 pN and 280 pN.

### The critical velocity defines a range of unfolding forces in titin

**Figure 2:**
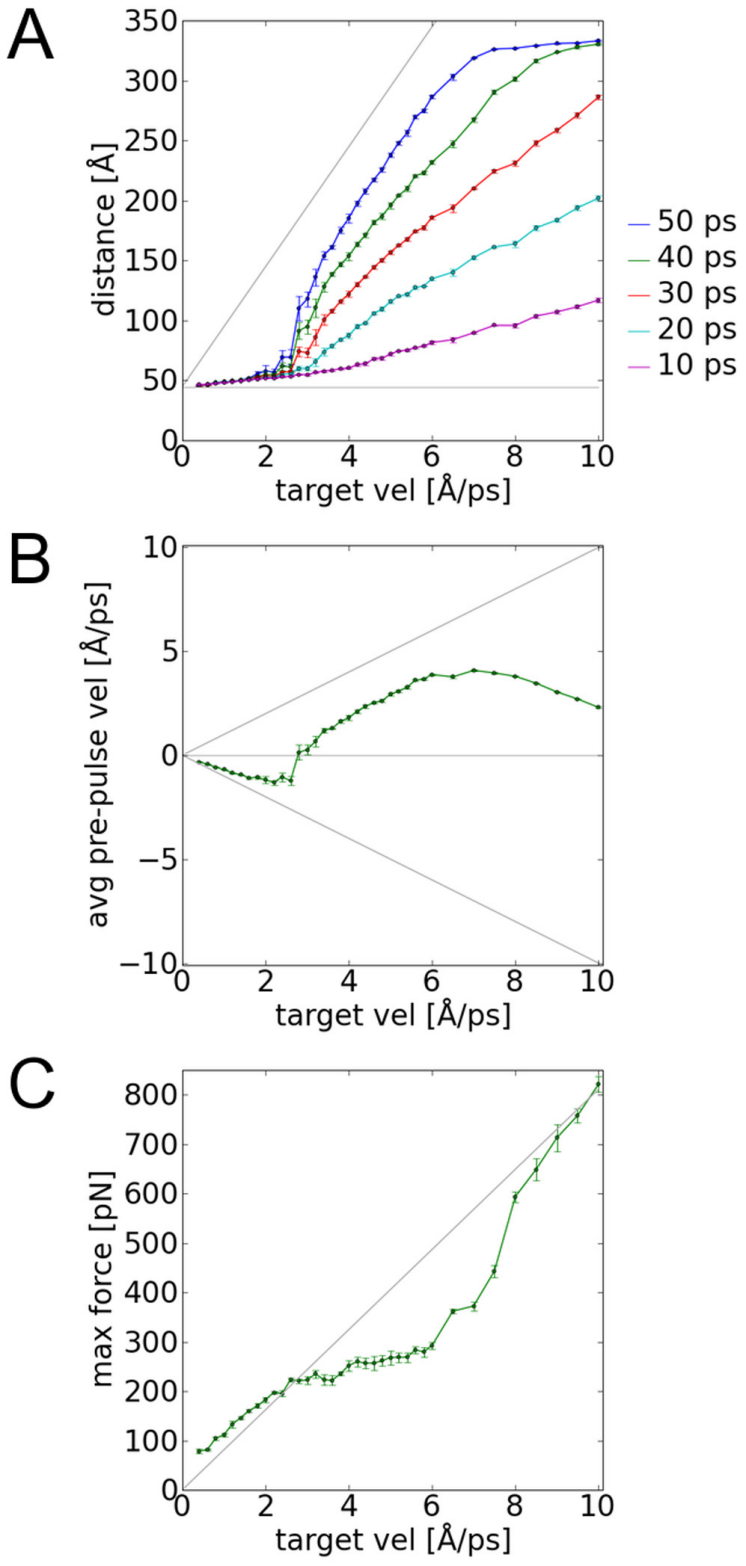
Analysis of the response of I27 to constant-momentum pulling over a range of target velocities. (A) In the distance response curves, the points along each column represents the distance evolution of the trajectory for a given target velocity. If the last point approaches the gray dotted line, the protein is unfolding at the target velocity rate. Otherwise the protein is trapped by an unfolding barrier. (B) In the velocity response curves the averaged pre-pulse velocity is plotted for each trajectory. Negative values means the protein is trapped in an intermediate or is completely extended. When the protein is unfolding without barriers, the values approaches the positive dotted curve. (C) In the fitted force response curves, the forces can be compared to the theoretical maximum force (2MV_target_) indicated by the gray line. When the system is trapped or completely extended, the maximum force is close the the theoretical maximum. When the system is unfolding with no barriers, the maximum force plateaus at the unfolding force of the protein.

Given that applying constant-momentum PUFF to the I27 domain produces a different response at different target velocities, the system can be characterized by performing simulations over a range of target velocities (Figure 2A). There are four regimes of response. In the saturated velocity range V_target_>8.0 Å/ps, the protein I27 has completely unfolded to the fully extended state within 50 ps. In the constant momentum range, 2.6 Å/ps<V_target_<8.0 Å/ps, the protein is unfolding near the target velocity rate. In the intermittent range, 1.4 Å/ps<V_target_<2.6 Å/ps, the protein is just beginning to unfold, but not at the full rate, and there is a range in which the protein fails to unfold.

We can derive a relationship between the target velocity and the maximum force, when the unfolding is impeded, i.e. for simulations where V_target_<2.6 Å/ps. In these simulations, the average pre-pulse velocities are negative, with a magnitude almost equivalent to the target velocity (Figure 2B). This is indicative of the system reflecting away from the edge of a potential well, where a positive force is needed to be continuously applied to bring the system to the target velocity (Figure 2C). To a good approximation then, the velocities fluctuate between ±V_target_. When the system is at -V_target_ the change in momentum required to bring the system back to +V_target_, is ΔP = M ΔV = M (+V_target_ - -V_target_)  = 2 M V_target_ . Since this an instantaneous event, the change in momentum is equivalent to the applied force F_max_ = 2 M V_target_.

This relationship should hold for target velocities where the unfolding is impeded such as the case of V_target_ = 1.0 Å/ps (Figure 1E–G). The averaged pre-pulse velocities is −0.7 Å/ps, which has a magnitude close to the target velocity of 1.0 Å/ps. From the equation, the theoretical maximum force is F_max_ = 2 M V_target_ = 82 pN. This compares well with the F_max_ = 92 pN deduced from the force-time curve (Figure 1G). For another comparison, the theoretical curve for maximum force F = 2 M V_target_ is plotted against the maximum force found in the simulations for a range of target pulling velocities (Figure 2C). For V_target_<2.6 Å, the simulated values lie on the theoretical curve. At greater target velocities, the simulated values veer off the theoretical curve, as at these velocities, the protein unfolds without impedance. In the saturated range, V_target_>8.0 Å, the protein is completely unfolded and resists the pulling. Therefore the simulated values rises back up to the theoretical curve.

The critical force required to unfold the protein without barriers can be derived by an analysis of these simulations. This is defined by the critical velocity between the intermittent range and constant-velocity range, giving a critical velocity of V_critical_ = 2.6 ⊕/ps. Above this target velocity, the maximum forces applied by PUFF are consistently capable of taking the system out of the well. Given the pulling mass of m = 224 Da = 41 pN⋅ps^2^/Å, this gives F_unfolding_ = 213 pN, which is in excellent agreement with the measured value of 180 pN [15]. Another estimate for the critical force (218 pN) can be deduced from the point on the force-response curve (Figure 2C) where the simulated values deviates from the theoretical curve.

### The unfolding intermediate of titin in longer simulations

**Figure 3:**
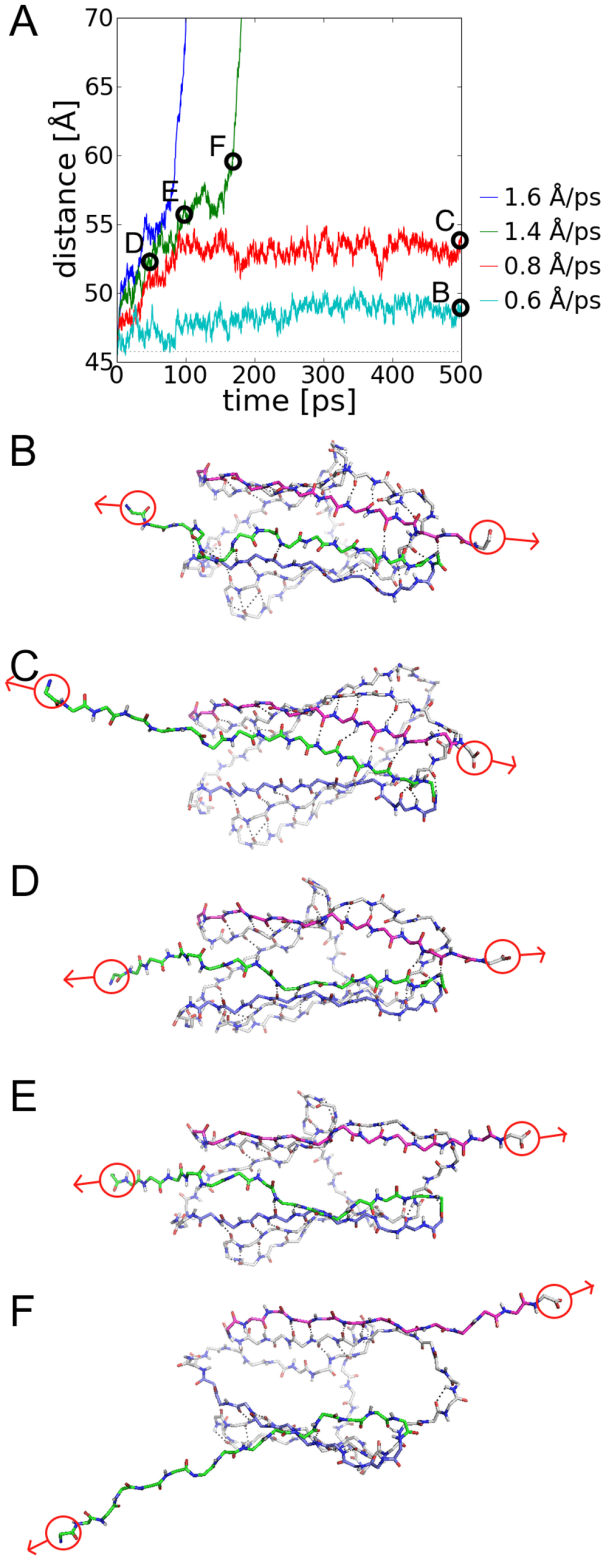
Unfolding intermediates of I27 detected in long simulations of 500 ps. (A) The evolution of the end-to-end distance of the trajectories from the initial state. The trajectory at V_target_ = 0.6 Å/ps (cyan trace) is trapped in the folded state. At V_target_ = 0.8 Å/ps (red trace), the system is trapped in an unfolding intermediate. At higher velocities (green trace, V_target_ = 1.4 Å/ps), the system works through a kinetic barrier before unfolding without impedance. The following snapshots show the key backbone hydrogen bonds between β-strand-A (green sticks), β-strand-G (purple sticks) and β-strand-B (blue sticks). (B) The last snapshot of the trapped trajectory (cyan trace, V_target_ = 0.6 Å/ps), with hydrogen bonds intact between β-strands-A, B – G. (C) The last snapshot from the trajectory trapped in the unfolding intermediate (red trace, V_target_ = 0.8 Å/ps) where the hydrogen-bonds between β-strand-A' and B are broken. The following snapshots are from a trajectory that unfolds through the kinetic barrier (green trace, V_target_ = 1.4 Å/ps): (D) three hydrogen-bonds between β-strand-A and G are broken; (E) all hydrogen bonds between β-strand-A and G are broken; and (F) the protein can now unfold without kinetic barriers at a constant velocity.

In the previous section, a series of short 50 ps trajectories were analyzed. Whilst it is clear that for constant-momentum with large target velocities, the I27 domain unfolds without barriers, it is not clear if the behavior in the intermittent range is a consequence of short simulations. To investigate this further, a series of PUFF simulations were performed with target velocities less than 2.6 Å/ps with a much longer simulation time of 500 ps (Figure 3A). From the distance-time trace of these simulation, it can be seen that some simulations appear to be trapped in a somewhat expanded intermediate state (red in Figure 3A), whilst others unfold completely (blue and green in Figure 3A). This trapped state can be compared to an experimentally characterized unfolding intermediate [15].

At a very small constant-momentum of V_target_ = 0.6 Å/ps, the end-to-end distance hardly changes over 500 ps (magenta in Figure 3A ). The maximum force found in this simulation is F_max_ = 57 pN, which is clearly insufficient to unfold the protein. In the last snapshot of this trajectory (Figure 3B), all key backbone hydrogen-bonds in the structure remain intact. Unlike conventional MD simulations, a PUFF force with a low target velocity actually stabilizes the folded state. In a conventional steered MD simulation, there is always the possibility that in a long enough time frame, a spontaneous fluctuation may unfold the protein. In a PUFF simulation, however, if a spontaneous fluctuation imparts a velocity much larger than 0.6 Å/ps, this will be damped immediately at the beginning of the next pulse, when the velocity will be set back to 0.6 Å/ps.

In a constant-momentum simulation at a slightly higher target velocity of V_target_ = 0.8 Å/ps, the protein is trapped in an unfolding intermediate at an extension of 10 Å (red in Figure 3A). The last snapshot of this simulation shows that the hydrogen bonds between β-strands A' and B are broken resulting in an extension of 10 Å (Figure 3C). The stability of this state for the last 400 ps suggests that 67 pN only provides enough force to break these hydrogen bonds. The value of 67 pN is close to the measured value of 100 pN for the intermediate [16].

At a constant-momentum simulation with V_target_ = 1.4 Å, the protein unfolds at the same rate as the target velocity after ∼170 ps (green in Figure 3A), but at earlier times there is a region where the protein is only slowly unfolding (marked by D, E and F in Figure 3A). At the beginning of this slow unfolding region at 50 ps, snapshot D (Figure 3D) shows that the protein has already reached the intermediate state, where the hydrogen bonds of A' and B are broken. Subsequently all hydrogen bonds between β-strands A and G break (Figure 3E – F), which constitutes the kinetic barrier from the intermediate to the unfolded state. At higher target velocities, such as V_target_ = 1.6 Å/ps (blue in Figure 3B), there is faster progression through the kinetic barrier.

By applying relatively low forces, it has been possible to identify an unfolding intermediate, and characterize the key structural transitions leading to the unfolding of this intermediate. To reach the intermediate from the folded state requires forces in the range of 57–67 pN to break the hydrogen-bonds between β-strands A' and B, which leads to an extension of 10 Å. Forces greater than 67 pN will break the barrier between the unfolding intermediate and the unfolded state due to the breaking of the hydrogen-bonds of β-strands A and G. This provides a lower bound to the kinetic barrier.

An upper bound to the kinetic barrier is given from the previous section, where a force of 213 pN was found to be sufficient to unfold the protein without impedance, where the hydrogen bonds between β-strands A and G can all be broken simultaneously. Below this value, in the range 67–213 pN, the forces can only break the hydrogen bonds sequentially between β-strands A and G. This range defines the kinetic unfolding barrier where unfolding is velocity dependent. This range of forces compares favorably to the experimental values of 60–150 pN derived for the folding intermediate [15]. As well, this range of forces also matches the range of forces 50–300 pN measured in the velocity-pulling AFM experiments [13].

### The mechanical unfolding of e2lip3

**Figure 4:**
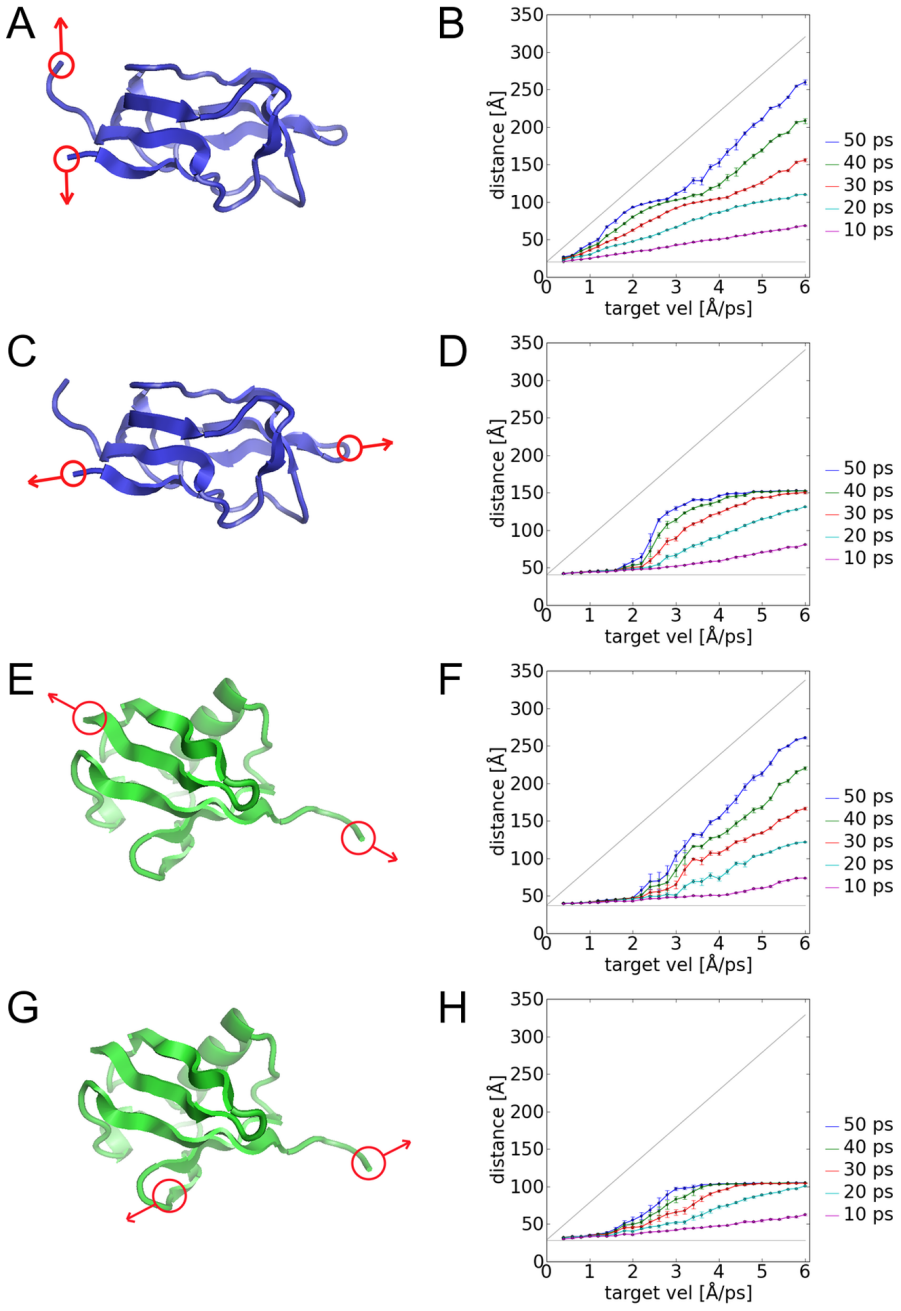
The constant-momentum simulations for the different pulling geometries of e2lip3 and ubiquitin. The pulling geometries are (A,B) N-C pulling in e2lip3, (C,D) N-41 pulling in e2lip3, (E,F) N-C pulling in ubiquitin, and (G,H) 48-C pulling in ubiquitin. The left column shows the schematic whilst the right column shows the distance-response curve as explained in the captions for Figure 2(A). By identifying where the major drop-off in distance response occurs, we can identify the critical target velocity, from which we can derive a critical unfolding force.

A strong test for PUFF is the ability to accurately model the differences in the unfolding forces for the same protein with different pulling geometries. AFM experiments were conducted on e2lip3 [21] where the pulling forces were applied between either the N- and C-termini (N-C pulling) (Figure 4A); or between the N-terminus and a prosthetic group attached to residue 41 (N-41 pulling) (Figure 4C) . The topology of e2lip3 places the N-terminus adjacent to the C-terminus as part of an antiparallel β-sheet, resulting in a negligible unfolding force in the N-C pulling experiment. By contrast, the N-41 pulling of e2lip3 results in a pulling geometry more like the titin experiments where the pulling forces would be parallel to a β-sheet [13]. Consequently, N-41 pulling resulted in a sharp force-extension curve with a peak at F_unfold_ = 182±5 pN. In contrast, N-C pulling resulted in a negligible response curve and probably falls below the force range that can be reliably applied by AFM (<15 pN). These differences have been explored in Steered MD simulations, where, although the differences were qualitatively captured, the magnitude of the simulation forces (∼400 pN for N-41 pulling, and ∼200 pN for N-C pulling) were much larger than the measured forces.

We applied PUFF with the velocity analysis for both pulling geometries of e2lip3. In the N-C pulling of e2lip3 (Figure 4A), the PUFF simulation was able to maintain the target velocity for all target velocities, indicating that there was only a negligible force barrier (an upper bound is F_unfold_<2 M V_smallest_ = 2×44×0.4 = 35 pN) (Figure 4B). In comparison, in the N-41 pulling (Figure 4C), there was a critical velocity of V_critical_ = 1.8 ⊕/ps (Figure 4D). Given a mass of 262 Da = 44 pN⋅ps^2^/Å for the anchor residues, this gives an unfolding force of F_unfold_ = 2 M V_critical_ = 2×44×1.8 = 158 pN, which is close to the measured value of 182±5 pN [21].

### The mechanical unfolding of ubiquitin

A similar AFM pulling experiment was conducted on ubiquitin [22], where two different pulling geometries were applied: that between the N- and C- termini (N-C pulling) (Figure 4E); and that between a prosthetic group attached to residue 48 and the C-terminal (48-C pulling) (Figure 4G). The topology of ubiquitin in terms of N-C pulling is similar to titin, resulting in a large barrier to unfolding with a measured unfolding force of F = 203±35 pN. In the pulling with the alternate geometry of 48-C pulling, a smaller critical force of F = 85±20 pN was measured. Simulations with Steered MD resulted in much larger forces of ∼2000 pN [22].

We applied PUFF with the velocity analysis to ubiquitin. In the N-C pulling (Figure 4E), there was a critical velocity of V_critical_ = 2.6 ⊕/ps (Figure 4F). Given a mass of 206 Da = 34 pN⋅ps^2^/Å for the anchor residues, this gives an unfolding force of F_unfold_ = 2 M V_critical_ = 2×34×2.2 = 177 pN, which is close to the measured unfolding force of 203±35 pN [21]. In the 48-C pulling (Figure 4G), the much lower critical velocity of V_critical_ = 1.6 ⊕ (Figure 4H). Combined with the pulling mass of 202 Da = 34 pN⋅ps^2^/Å results in a critical unfolding force of 109 pN. This also compares favorably with the experimental value of 85±20 pN.

### Choosing the relaxation time between pulses

**Figure 5:**
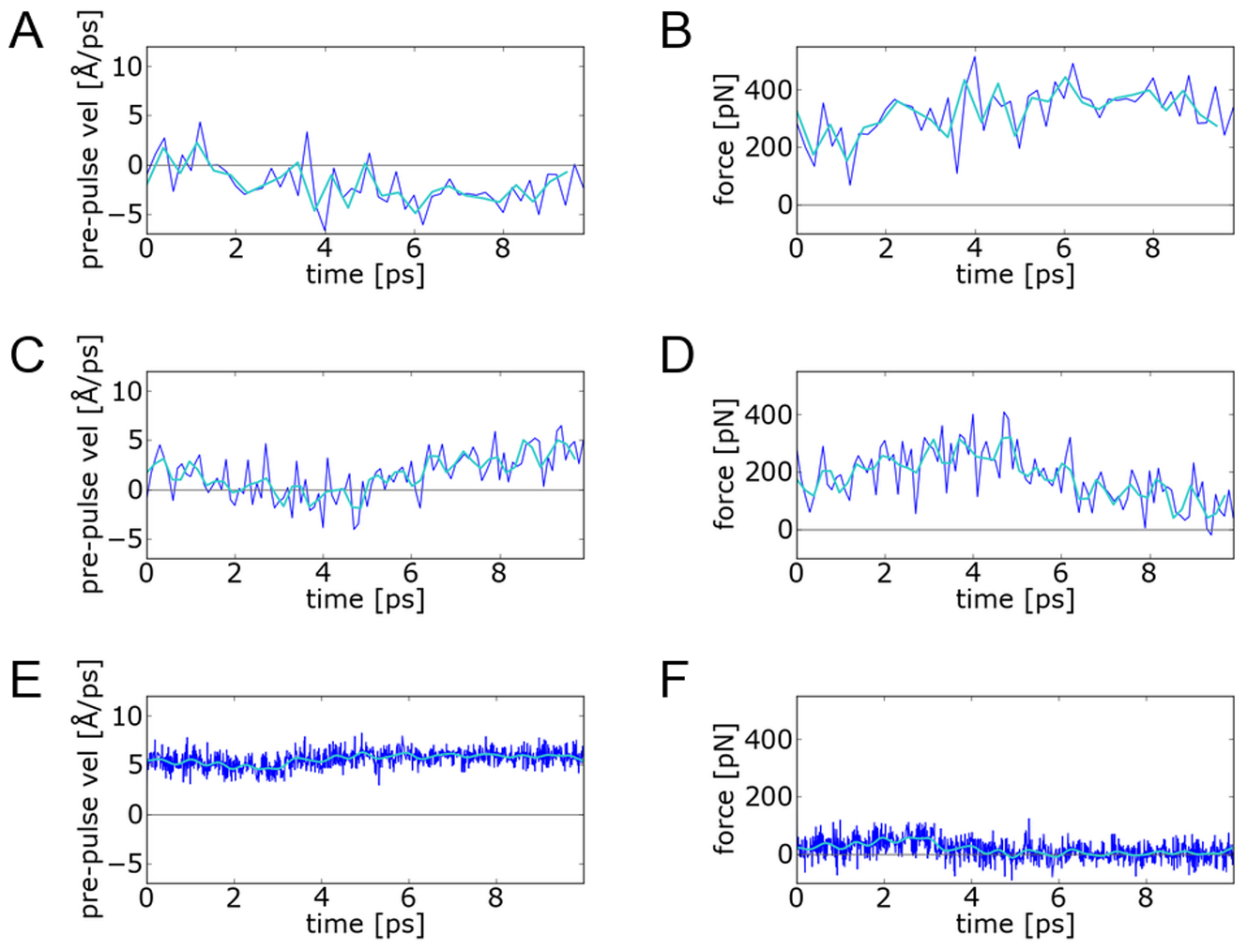
The effect of different relaxation times on trajectories of titin pulled at 6.0 Å/ps for 10 ps. The first row shows a trajectory with a large relaxation time of 200 fs, with plots of (A) the pre-pulse velocities and (B) the force response. The pulses here are applied so infrequently that the system cannot escape out of the folded state, indicated by the negative pre-pulse velocities. The second row corresponds to the default relaxation time of 100 fs, with (C) pre-pulse velocities that oscillates around zero before rising to the target velocity of 6.0 Å/ps, and (D) forces rising to a peak and then falling. The third row corresponds to a small relaxation time 10 fs, where (E) the target velocity is easily maintained with small fluctuations, and the system is never allowed to relax to negative values, which corresponds to (F) a very small force response.

The choice of relaxation time between pulses plays a crucial part in defining the response to PUFF pulling. To study the effect of different relaxation times, we repeated the titin pulling at 6.0 Å/ps for 5 ps with relaxation times of 200 fs, 100 fs and 10 fs (Figure 5). At large relaxation times (200 fs; Figure 5A – B), the protein is effectively trapped as the system remains trapped in a minimum that is much deeper that the supplied force. Smaller relaxation times (4 fs; Figure 5E – F) actually reduces the fluctuation in the system At this relaxation time, the system has not yet relaxed sufficiently by the next pulse and PUFF only needs to generate a small force to maintain the system at the desired velocity.

Thus, to observe natural fluctuations in PUFF, a certain amount of relaxation time is necessary. A good amount of fluctuation corresponds to a trajectory where the pre-pulse velocities oscillate between positive and negative values, as the simulation explores the full extent of a local minimum. In such a situation, the force calculated by PUFF can characterize the extent of the energy well. Heuristically, we have found that a value of 100 fs (Figure 5C – D) generates trajectories where the velocities oscillate between positive and negative values. Longer relaxation times may have to be used in larger systems to allow this characteristic oscillation in velocities.

## Discussion

Although MD simulations are beginning to breach the one microsecond barrier, there is still a long way to go before large-scale conformational changes can be directly observed. In the meantime, there remains a need for techniques that apply external forces to explore conformational changes in a practical amount of time. Here, the focus is on systems where proteins are mechanically pulled to induce unfolding. Such systems have been explored by AFM experiments, which provide detailed force measurements that constitute a rigorous test of the accuracy of any computational force-generating methodology.

In the previous literature, most of the focus has been on constant-velocity AFM pulling experiments, which generate a characteristic saw-tooth force profile over the extension of the protein [13]–[14]. Sufficiently slow pulling velocities (∼10^−8^ Å/ps) are used to allow accurate closed-loop control. A linear relationship has been observed between the maximum force and the pulling velocity over a range of pulling velocities. More recently, force-clamp AFM experiments have provided an alternative view of the unfolding force profile [15]. In force-clamp AFM experiments, constant forces are generated. For certain range of forces, the protein unfolds to a specific end-to-end distance, corresponding to different stages of unfolding. The force-clamp forces provide a different characterization of the unfolding landscape of titin.

Steered MD simulations have been used to explore constant-velocity motions where pulling velocities (1 Å/ps) 10^8^ much faster than the AFM experiments are used to generate sufficient motion within a reasonable timescale (less than a nanosecond). Although Steered MD simulations have reproduced the linear dependency between forces and pulling velocities, the simulated force fluctuations were much larger than expected from the AFM experiments. This has been found using both implicit [21] and explicit solvent [18]. As such the discrepancy cannot be attributed to hydrogen bonding with explicit solvent. The most likely source is the elasticity of the harmonic springs used to generate the forces in Steered MD.

In contrast, the PUFF methodology generates forces directly without the need of harmonic springs. Although both PUFF and Steered MD simulations are parameterized by a target velocity, the target velocity in PUFF is conceptually different to the target velocity in Steered MD. In Steered MD, once the harmonic spring restraints are set to the target velocity, the instantaneous velocities are allowed to fluctuate wildly, whilst the overall velocity, averaged over a time-scale larger than the response of the harmonic spring, is maintained to a fixed value. In contrast, in a PUFF simulation, it is the instantaneous velocity that is fixed at the beginning of every pulse, which constrains the instantaneous momentum. If the applied momentum is insufficient to break out of a local minimum, then the protein gets trapped.

In PUFF simulations, then, the forces are capped, which can retard the overall motion, whilst in Steered MD, forces can fluctuate wildly, but the overall motion is fixed. Conceptually then, the PUFF simulations are closer to the force-clamp AFM experiments, which measure a range of static forces for different levels of unfolding. Simply by noting whether a PUFF simulation unfolds at the target velocity or at an impeded rate or not all, we can calculate a corresponding range of forces, where the range of forces from PUFF agree well with the force-clamp AFM experiments for titin. As PUFF does not model the kinetics of unfolding at a fixed velocity, it is not expected to model the relationship between force and pulling velocity found in constant-velocity AFM experiments. However, for purposes of comparison with other proteins, we assume that the force measured in constant-velocity AFM experiments falls near the value where the protein unfolds without impedance in the PUFF simulations. As such, the PUFF simulations produce values that agree well with the AFM pulling experiments of e2lip3, and ubiquitin.

Another advantage of PUFF is that the relaxation period after the pulse allows the protein to respond to the applied forces in qualitatively different ways. We can use the trajectories where the I27 domain is trapped to identify unfolding intermediates and reproduce the range of forces that determines the unfolding intermediate. In previous Steered MD simulations, the I27 unfolding intermediate was also identified using constant-force pulling restraints [16]. However, such constant-force restraints in Steered MD can only be used to study intermediates at small extensions because for larger motions, it is difficult to rationalize the stability of the constant-force restraints. Instead, constant-velocity restraints must be used for large motions in Steered MD, but they result in highly inaccurate force values [18], [19]. In contrast, with PUFF simulations, the same type of constant-momentum simulation can be used to identify both folding intermediates and critical unfolding forces. Given the varied response with the same type of simulation, we can extend PUFF to study protein deformations where there is a differential response of the protein to the applied force. Indeed, we have already been able to generate such differential conformational responses using local rotational forces [20].

The tradeoff in PUFF is in the overhead of implementing the protocol within standard MD packages. In PUFF, the simulations are performed in pulses outside the MD simulations, which require PYTHON scripts to make calculations between each MD run of the pulses. However, this allows the PUFF technique to be easily ported to other MD packages. As well, it becomes much easier to implement other more complex forces (we are currently exploring domain-domain interactions).

Currently, PUFF is implemented in AMBER using a GB/SA implicit solvent potential. As the implicit potential used in PUFF is able to derive realistic force values, this suggests that the main component of the force barrier are the internal hydrogen bonds. However, the derivation of the complete free-energy profile requires the accurate modeling of kinetics, especially the role of explicit waters. In previous Steered MD studies of the unfolding of titin, it was that found that hydrogen bonding with explicit solvent waters plays a key role in defining the kinetics [23]–[24]. In particular a reasonable estimate of the unfolding barrier was derived from the first mean passage times. It would thus be useful to extend the PUFF simulations to include explicit solvent. Nevertheless, Steered MD consistently overestimate force fluctuations due to the harmonic springs. To explore other thermodynamic parameters such as the work function, trajectories with better force values will be needed. By removing the dependency on harmonic springs, the adaptive forces of PUFF can generate trajectories with less force fluctuations at faster velocities and shorter simulation times.

## Methods

The MD simulations are performed by the AMBER package. The AMBER96 force-field was used with the GB/SA implicit solvent. The proteins were pre-equilibrated to 300 K for 100 ps using a Langevin thermometer with a friction coefficient of γ = 5 s^−1^.

The pulses were carried out by performing constant energy MD simulations of 100 fs with a time-step of 1 fs. Between each pulse, the simulations are stopped, where the coordinates and velocities are read from the restart files by PYTHON scripts. The velocities in the system are first scaled to 300 K. Then modified velocities are generated and applied to the system. The new system are written to new restart files. The simulations are restarted for the next 100 fs pulse. The modified velocities represents the applied forces. One of the features of PYTHON is that it allows the use of dynamic and functional programming techniques that makes it quite easy to implement forces in PYTHON. The library for the PYTHON code implementing the PUFF protocol can be downloaded from http://boscoh.com/puff.

To generate repeats of the pulling simulations, starting conformations were taken from different points of the 100 ps equilibration: the 100, the 90, 80, 70 and 60 ps conformations.

The simulations are defined by a target velocity V_target_. At the beginning of the simulations, two sets of residues (group1 and group2) are chosen to be the anchor points of the pulling. Between each pulse, the axis direction between the center of mass of group1 and group2 is first calculated as **N**_12_ (vectors are in bold). Then the velocity of the center mass of both groups are calculated as **V**_1_ and **V**_2_. From the relative velocities of group2 from group1 **V**_12_ = **V**_2_ - **V**_1_, the relative velocity is defined along the axis V_12,axis_. We can then extract the magnitude V_12,axis_.

To force the system to move at a given target velocity V_target_, the change in velocity is ΔV = V_target_ - V_12,axis_. Since the force is applied at an instant between pulses, time intervals are not necessary, and the acceleration vector is set to **A** = ΔV×**N**_12_ in the direction of the axis between the center of mass **N**_12_. Since we want the motion to be equal and opposite we apply 0.5×**A** to every atom in group 2, and −0.5×**A** to the atoms in group1. The force is calculated as F = M×A where M is the mass of group1 and group2.
